# Distinct Neuropsychological Correlates of Apathy Sub-Domains in Multiple Sclerosis

**DOI:** 10.3390/brainsci13030385

**Published:** 2023-02-23

**Authors:** Simona Raimo, Mariachiara Gaita, Antonio Costanzo, Daniele Spitaleri, Gabriella Santangelo

**Affiliations:** 1 Department of Medical and Surgical Sciences, University “Magna Graecia” of Catanzaro, 88100 Catanzaro, Italy; 2 Department of Psychology, University of Campania “Luigi Vanvitelli”, 88100 Caserta, Italy; 3 Neurology Unit “San Giuseppe Moscati”, Hospital Avellino, 83100 Avellino, Italy

**Keywords:** multiple sclerosis, apathy, behavioral disorders, cognitive dysfunctions

## Abstract

Background: Apathy is relatively frequent and significantly associated with clinical and cognitive outcomes in Multiple Sclerosis (MS), even if previous research has produced mixed results. This varied picture could be due to most studies treating apathy as a unitary construct, despite the evidence showing that apathy is a multifaceted syndrome including three different sub-domains (i.e., cognitive, affective, and behavioral). This study aims to investigate the neuropsychological correlates of apathy fractionated into its three sub-domains in participants with MS. Methods: Eighty-five participants with MS underwent a comprehensive neuropsychological battery. The severity of apathy symptoms was assessed by the self-report version of the Apathy Evaluation Scale. Results: Correlational analysis showed that cognitive apathy sub-domain scores had a high correlation with the performances obtained at cognitive tests tapping into inhibitory control (i.e., IML and Strop test-interference task), whereas the affective apathy sub-domain scores had a high correlation with the performances obtained at cognitive test tapping into the use of executive functions in visuospatial abilities (i.e., Clock Drawing Test). Moreover, linear regression analysis results showed that the cognitive apathy sub-domain scores predicted executive functioning domain scores and that the cognitive and affective apathy sub-domains scores predicted visuospatial abilities domain scores. Conclusion: These results confirm that apathy is a multidimensional concept with important neuropsychological correlates, visible only when it is fractionated into its sub-domains.

## 1. Introduction

Apathy constitutes a pathological reduction in self-generated voluntary and purposeful acts, that reflects dysfunctions in cognitive, affective, and behavioral processes relating to the execution, planning, and control of goal-directed behavior [1,2,3,4]. Previous studies show that apathy is one of the most frequent non-physical symptoms of Multiple Sclerosis (MS), with a prevalence ranging from 20% to 50% [3,4,5,6,7,8], and would be significantly associated with neurological disability, fatigue, caregiver distress, and neuropsychological dysfunctions [7,8,9,10,11]. Indeed, apathy has been shown to be significantly associated with alterations in tests that tap into executive functions, particularly in those assessing inhibitory control [8]. This finding underlines how apathetic symptomatology may be associated with dysexecutive syndrome [12]. This association has been highlighted not only in patients with MS but also in other neurodegenerative disorders (e.g., Parkinson’s Disease), where the development of apathy was predicted by an impairment of inhibitory control over time [13]. Furthermore, longitudinal studies have shown that apathy may be a neurobehavioral marker predictive of cognitive decline in MS [10,11]. However, despite its high prevalence and negative consequences, apathy is still a mistreated neuropsychiatric syndrome in MS clinical practice with no standard management approaches [14].

Additionally, studies investigating the neural substrates of apathy in neurologically intact subjects highlight the clinical and research importance of considering apathy as a multifaceted syndrome, since processes disabled in apathy, such as those involved in self-initiative, emotional expression, feeling, and volition, would involve different cortical and subcortical brain areas [15,16]. In particular, apathy would appear to be associated with a reduction in grey matter volume in the anterior cingulate, ventromedial orbitofrontal cortex, and insula cortex. However, the insula would play a role in the perception of emotions, integrating sensory and interoceptive signals in relation to the motivational state [17], whereas the ventromedial orbitofrontal cortex and the anterior cingulate would play an important role in higher-order functions such as decision-making, learning, and attentional processes that regulate actions and guide behavior [18].

These findings highlight the clinical and research importance of considering apathy as a multifaceted syndrome.

Indeed, diagnostic, clinical, and neuroimaging criteria [2,15,16,19,20,21] differentiated apathy into three distinct sub-dimensions: i. affective apathy, characterized by diminished emotional expression/responsiveness, associated with dysfunctions in the mesocorticolimbic dopaminergic pathway and lesions in the orbital–medial prefrontal cortex and structures of the basal ganglia; ii. cognitive apathy, characterized by a reduction in interests, loss of initiative, inactivity in goal-directed behavior, associated with dysfunctions in the cortical cholinergic pathway, and lesions in the dorsolateral prefrontal cortex and sub-regions within the basal ganglia; and iii. behavioral apathy, characterized by a marked reduction in initiating and sustaining autonomous activities related to daily self-care actions, associated with low activity in the bilateral insula.

So far, only a few neuropsychological studies [22,23] take into account the multidimensional nature of apathy and acknowledge the importance of treating these domains distinctly in investigating the neuropsychological profiles that accompany them, finding that cognitive apathy would be mainly involved in executive functioning, and affective apathy in emotion perception. Thus, the main aim of this study is to identify the neuropsychological correlates of the cognitive, behavioral, and affective sub-domains of apathy in MS, hypothesizing that each of these three sub-domains would have distinct neuropsychological correlates.

## 2. Materials and Methods

### 2.1. Participants

One hundred and five patients with a diagnosis of MS, according to diagnostic criteria [24], were recruited. Patients were consecutively enrolled at the Multiple Sclerosis Center of Moscati Hospital in Avellino, Italy, and they were excluded from the present study based on the following criteria: (i) major neurocognitive disorder (according to the Diagnostic and Statistical Manual of Mental Disorders-DSM-5 [25]); (ii) general intellectual decline as defined by a Mini-Mental State Examination score (MMSE) in the normal range [26] lower than 23.8 according to Italian norms [27]; (iii) severe neurological disability according to a score higher than 7 on the Expanded Disability Status Scale (EDSS; [28]); (iv) history of psychiatric illnesses except for the diagnosis of apathy; (v) history of head trauma, neurologic diseases, or alcohol or drug abuse; (vi) other autoimmune diseases; and (vii) non-native Italian speaking subjects. For each patient, demographic data was recorded, such as age, sex, and level of education, and clinical aspects, such as disease duration, age at disease onset, level of disability (EDSS), and current pharmacological treatment.

The study was performed following the ethical standards laid down in the 1964 Declaration of Helsinki. It was approved by the local ethics committee, and participants gave written informed consent before participation.

### 2.2. Neuropsychological Assessment

All participants were administered a comprehensive neuropsychological battery that investigated the following cognitive functions:

i. global cognitive functioning by means of the Italian version of the MMSE [27], consisting of eleven questions tapping into temporal and spatial orientation, immediate and delayed verbal memory, language, attention, and praxis abilities, with a total score ranging from 0 to 30 according to the number of correct responses.
ii. verbal memory by means of the immediate and delayed recall conditions of the Rey Auditory Verbal Learning Test (RAVLT; [29]), consisting of a list of 15 words not semantically related to each other that participants are asked to remember both immediately, after the words are read for five times (learning recall), and, subsequently, after 20–30 min (delayed recall), with two total scores ranging from 0 to 75 for the learning recall, and from 0 to 15 for the delayed recall corresponding to the number of words correctly recalled;
iii. visuospatial memory by means of the delayed recall of the Rey–Osterrieth Complex Figure Test, (ROCF, [30]), asking participants to draw from memory a previously presented complex figure composed of 18 elements, with the total correct score ranging from 0 to 36, according to the number of elements correctly drawn and placed (a score of 2 points may be awarded for each element);
iv. visuospatial abilities by means of: the Raven’s Colored Progressive Matrices (RCPM; [29]), a non-verbal intelligence task tapping into logical reasoning on visuospatial material, with the total score ranging from 0 to 36 according to the number of matrices correctly completed; the Constructional Apraxia Task (CAT; [31]), tapping into construction skills by asking the participant to copy seven figures, with a total score ranging from 0 to 14 given by the number of figures correctly reproduced (a score of 2 points may be awarded for each figure); and the ROCF copy, asking participants to copy a complex figure, with the total score ranging from 0 to 36 given by the number of correctly reproduced elements;
v. executive functioning by means of: the Clock Drawing Test (CDT; [32]), asking participants to place numbers on a printed circle as if depicting the face of a clock and then to draw the clock hands indicating a certain time (i.e., ten minutes past 11 o’clock), with the total score ranging from 0 to 10 given by the presence of the correct numbers and by the spatial accuracy of both the numbers and the hands; the Trail Making Test (TMT:B-A; [33]), consisting of two parts (A and B) asking participants to as quickly and accurately as possible connect a series of 25 circled and scattered numbers in ascending order (part A) and then to alternate numbers and letters in ascending/alphabetical order (1-A-2-B-3, etc.; part B), with the total score corresponding to the difference calculated in seconds taken by the participants to complete the part B and A of the task (part B-part A); Stroop test-interference task [34], tapping into the ability to inhibit cognitive interference by asking participants to name the color of the ink with which the color word is written (e.g., the word ‘violet’ printed with brown ink), with the total score given by the number of correct answers in 30 s; the Inverse Motor Learning Test (IML; [31]), tapping into the ability to inhibit imitation and perseveration by asking participants to inhibit a behavior shown by the examiner (e.g., every time the examiner raises his or her hand open, showing the palm, the participant must raise it closed, showing the fist, and vice versa) with the total score ranging from 0 to 24 given by the number of gestures correctly performed; the phonological verbal fluency task [29], tapping into lexical ability by asking participants to recall as many words as possible according to the initial letter (F, A, and S) within 1 min, with the total score given by the sum of the correctly retrieved words in all three conditions; and the semantic verbal fluency task [31], tapping into the participant’s ability to recall as many words as possible that belong to a certain semantic category (colors, animals, cities, and fruit), within 1 min, with the total score given by the average of the correctly re-enacted words for each category.

The severity of apathy symptoms was assessed by the self-report version of the Apathy Evaluation Scale (AES-S; [1]), validated in participants with MS [6]. It consists of 18 items rated on a 4 Likert points scale (to mean “not at all true”, “slightly true”, “somewhat true”, or “very true”) based on the subject’s functioning during the previous 4 weeks and assessing three specific sub-dimensions of apathy: behavioral (i.e., reduction in goal-directed behaviors) by means of five items (2, 6, 10, 11, 12; e.g., “Someone has to tell me what to do each day”, “I get things done during the day”, “I am less concerned about my problems than I should be”); affective (i.e., blunted emotions) by means of two items (8, 14; e.g., “When something good happens, I get excited”); and cognitive (i.e., reduced number of intentions towards goal-directed behavior) by means of eight items (1, 3, 4, 5, 7, 9, 13, 16; e.g., “I am interested in things”, “Getting things started on my own is important to me”, “I am interested in having new experiences”). The remaining three items (15, 17, 18, e.g., “I have an accurate understanding of my problems”, 18 “I have the motivation”) belong to a group called “other” [1]. The total score ranges from 18 to 72, with higher scores meaning more severe apathy.

The severity of depressive symptoms was assessed by the Hamilton Depression Rating Scale (HDRS-17; [35]), validated in MS [36]. This version consists of 17 items and assesses depressive symptoms experienced in the last week. Some items (4, 5, 6, 12, 13, 14, 17) are rated on a 3-point Likert scale, ranging from 0 to 3 levels of severity, while others (1, 2, 3, 7, 8, 9, 10, 11, 15, 16) are rated on a 4-point Likert scale, ranging from 0 to 5 levels of severity. The total score ranges from 0 to 54, with higher scores indicating more severe depressive symptoms.

### 2.3. Statistical Analysis

To investigate the relationship between behavioral, affective, and cognitive sub-domains of apathy and performance on neuropsychological tests, we performed Pearson correlation analyses. Subsequently, to identify which sub-domain of apathy (i.e., behavioral, affective, cognitive) was able to predict significant alterations in the specific cognitive domains (i.e., memory, visuospatial, and executive), linear regression analyses were performed. In particular, each raw score of neuropsychological measures was converted into a z-score using the mean and standard deviation (SD) of normative data, and z-scores were summed to obtain a total score for the following cognitive domains: i. executive functioning, ii. visuospatial abilities, and iii. memory. In particular, the total executive function score was obtained from the sum of the z-score of the following tests: CDT, TMT:B-A, Stroop test-interference task, phonological verbal fluency task, semantic verbal fluency task, and IML. The total score of visuospatial abilities was obtained from the sum of the z-score of the following tests: RCPM, copying ROCF and CAT. The total score of memory was obtained from the sum of the z-score of the following tests: immediate and delayed recall conditions of RAVLT and delayed recall of ROCF. In the linear regression analyses, we used the total score of each cognitive domain and depressive symptomatology as a dependent variable and the sub-domain apathy sub-scores as independent variables.

Moreover, we performed regression analyses to test the effect of age on the three sub-domain apathy sub-scores, using the sub-domain apathy scores as dependent variables and age as the independent variable.

Although the value of *p* < 0.05 was considered statistically significant, the Bonferroni correction for multiple comparisons was applied. All statistical analyses were performed with the software SPSS (version 21; SPSS Inc., Chicago, IL, USA).

## 3. Results

In this study, we excluded 10 participants since they reported a deficit in global cognitive functioning, and another 10 participants because of severe disability. The final sample was then composed of 85 participants (16 males and 69 females) affected by remitting-relapsing MS. The demographic, behavioral, and clinical characteristics of the whole sample are summarized in Table 1.

Results of correlational analyses showed that the cognitive sub-domain of apathy scores was highly and significantly associated with performances obtained at the tests assessing inhibitory control (i.e., IML and Strop test-interference task). Moderate and significant correlations were also found between these cognitive tests (i.e., IML and Strop test-interference task) and the affective and behavioral sub-domains’ apathy scores. The scores of the affective sub-domain of apathy had high and significant correlations with the CDT, whereas the cognitive apathy sub-domain scores had moderate and significant correlations with the ROCF. Moreover, the scores of the behavioral sub-domain of apathy were moderately correlated to the HDRS scores, even if these correlations did not survive after the Bonferroni correction. See Table 2.

Results of linear regression analyses showed that the scores of the behavioral apathy sub-domain did not predict the memory (β = 0.192, t = 1.503, *p* = 0.136), executive functioning (β = −0.038, t = −0.316, *p* = 0.753), and visuospatial abilities (β = 0.144, t = 1.187, *p* = 0.238) domains’ scores, whereas the scores of the affective and cognitive apathy sub-domains significantly predicted the scores of the visuospatial abilities domain (β ≥ −0.250, t ≥ −2.024, *p* ≤ 0.046), and the scores of the cognitive apathy sub-domain also predicted executive functioning (β = −0.239, t = −2.077, *p* = 0.040) but not memory (β = −0.065, t = −0.536, *p* = 0.593). Moreover, none of the three sub-domain scores predicted depressive symptomatology (β ≥ −0.050, t ≥ 0.321, *p* ≤ 0.749). Finally, age significantly predicted affective apathy sub-domain scores (β = 0.337, t = 3.591, *p* = 0.001), cognitive apathy sub-domain scores (β = 0.389, t = 4.242, *p* ≤ 0.001), but not behavioral apathy sub-domain scores (β = 0.145, t = 1.469, *p* = 0.145).

## 4. Discussion

This study investigated the possible association between the cognitive, affective, and behavioral sub-domains of apathy and neuropsychological functioning in MS. We found that all the sub-domains of apathy scores significantly correlated with the performance on executive functioning. In particular, the cognitive apathy sub-domain was highly associated with the ability to inhibit control, and cognitive and affective apathy correlated significantly with the performance obtained in tests tapping into the use of executive functioning in visuospatial abilities. Any correlations between performance scores obtained by participants with MS on the tests assessing memory, verbal fluency, and reasoning and the sub-domains of apathy were found, thus assuring that overall cognitive dysfunction is not supposed to be related to apathy (i.e., memory or language disorders). Indeed, previous studies in clinical (i.e., stroke, [37]; Parkinson’s Disease, [38]; Mild Cognitive Impairment and Alzheimer’s Disease, [39]; Behavioral Variant of Frontotemporal Dementia, [40]) and non-clinical populations (healthy elderly, [41]) found that apathy was specifically related to impaired executive functioning, but not memory or language deficits.

Regression analysis results showed that executive functioning was significantly predicted by the cognitive sub-domain of apathy, whereas visuospatial abilities were significantly predicted by cognitive and affective sub-domains of apathy. Overall, these results confirm the link between apathy and executive dysfunctions in MS [6,8,10,11]; highlighting as higher cognitive apathy symptoms would predict more inhibitory control deficits. Accordingly, Levy and Dubois [2] named the cognitive sub-domain of apathy “cognitive inertia”, characterized by a reduction in goal-directed behavior due to deficits in the higher-order functions involved in the cognitive control of activities, such as task switching, planning, and working memory. From a neural point of view, a reduction in goal-directed behavior would, indeed, be associated with lesions of the dorsolateral prefrontal cortex (i.e., BA 9/46), ventrolateral (i.e., BA 12, 44, 45, 47), and frontopolar (i.e., BA 10) regions [42,43,44]. Additionally, the significant association between the affective sub-domain of apathy and the performance on the CDT would suggest an important mediating role of visuospatial functioning in the relationship between executive dysfunction and affective processing, as seen in previous studies [45,46].

Notably, the behavioral sub-domain of apathy did not predict any cognitive test scores. These results are in line with the view of Levy and Dubois [2], suggesting a replacement of the term behavioral apathy with the concept of an “auto-activation deficit”, characterized by difficulties in activating thoughts or initiating motor programs required to complete a task and mainly related to the activity of the mesolimbic and basal ganglia dopamine systems [47]. From a clinical perspective, patients tend to remain silently in the same place or position all day long, without taking any spontaneous initiative or speaking [48]. Thus, this dimension of apathy would not be a conceptually or theoretically valid correlate of cognitive functioning, but, rather, a correlate of affective states since would involve the reward system regulating motivational anticipation and evaluating the pleasure anticipation and effort costs of an activity that should be voluntarily undertaken [47]. Based on the above, it can be hypothesized that the moderate correlation of the behavioral sub-domain of apathy with depressive symptomatology may just be the reflection of a disturbed reward system (i.e., the mesolimbic dopamine system) involved in affective processes of ongoing behavior [49,50]. According to previous literature (i.e., [51,52]), such a result did not exclude a significant association with covert cognitive changes identifiable through electrophysiological methods (e.g., event-related potentials) or specific questionnaires validated to assess subjective cognitive impairments, which should be better investigated in future studies. Indeed, subjective cognitive impairment is commonly reported in MS in association with abnormalities of brain volume and activity [53] and with negative affectivity and stress levels. For the first time in a clinical sample, our results revealed that age would play a different role in the sub-domains of apathy, with older participants reporting higher levels of cognitive (i.e., diminished interest in new experiences and learning) and affective (i.e., diminished emotional responsivity and social interaction), but not behavioral (i.e., diminished initiative and drive) apathetic symptomatology. Overall, our findings suggest the importance of early intervention of apathy to promote cognitive functioning in MS. Apathy should be assessed and managed before the onset of cognitive impairment in MS, which may help them prevent all clinician-rated everyday problems (e.g., quality of life, caregiver distress, functional status) commonly associated with apathy.

Some limitations of this study should be revealed. First, gender-related differences in apathetic dimensions were not investigated due to the limited number of males (*n* = 16) in our sample, with MS being a female-dominated disease. Second, neuroimaging data could be crucial for revealing the neuroanatomical regions mainly involved in the different apathy subdomains and the associated cognitive deficits. Furthermore, we have not investigated other behavioral variables (e.g., Theory of Mind, decision-making) that are associated with the different sub-domains of apathy in other diseases [22,23]. Finally, we used a self-report scale validated in SM to assess the sub-domains of apathy as we were interested in investigating the neuropsychological correlates of the behavioral, cognitive, and affective apathetic symptoms. Future studies should be conducted to identify people with MS with distinct apathetic sub-domains (i.e., cognitive apathy, affective apathy, or behavioral apathy) to confirm our results with comparative analyses and address the unresolved issues.

## 5. Conclusions

In summary, we found that the three apathy sub-domains would have distinct cognitive correlates that could mask the treatment of apathy as a unitary syndrome. Indeed, considering that processes directing and sustaining human-motivated behavior are broad, ranging from those involved in rewards and punishments (i.e., behavioral apathy) to those that are engaged in executive control and planning (i.e., cognitive apathy) to those that initiate feelings (i.e., affective apathy), fractionating apathy symptomatology into its sub-dimensions could be a useful approach for a better understanding and better management of it in clinical practice. Future studies investigating specific processes for the execution of goal-directed behavior manifesting as distinct apathetic states, including patients affected by other forms of neurodegenerative disorders, could provide new insights into apathy and its neuropsychological and neural underpinnings.

## Figures and Tables

**Table 1:** Demographic, behavioral, and clinical variables (Mean ± Standard Deviation) of participants with MS (*n* = 85).

*Demographic Variables*	Mean ± SD	Range (Min–Max)
Age (years)	43.27 ± 11.1	21–68
Education (years)	12.47 ± 3.59	5–18
***Clinical variables***		
EDSS	3.28 ± 1.53	1–6
Duration of disease (months)	114.93 ± 88.26	10–432
Age at onset of disease (years)	9.53 ± 7.24	1–36
***Global cognitive functioning***		
MMSE	28.32 ± 1.99	20–30
***Behavioral Variables***		
AES	33.97 ± 8.97	19–56
AES-C	14.51 ± 4.51	5–27
AES-B	9.30 ± 3.11	5–22
AES-A	4.17 ± 1.45	2–8
HDRS	8.68 ± 4.71	0–16

Note. MS: Multiple Sclerosis; EDSS: Expanded Disability Status Scale; MMSE: Mini-Mental State Examination; AES: Apathy Evaluation Scale, AES-C: Apathy Evaluation Scale, a sub-scale score of cognitive apathy symptoms; AES-B: Apathy Evaluation Scale, a sub-scale score of behavioral apathy symptoms; AES-A: Apathy Evaluation Scale, a sub-scale score of emotional apathy symptoms; HDRS: Hamilton Depressive Rating Scale.

**Table 2:** Correlations between AES sub-domains and cognitive functions in participants with Multiple Sclerosis.

*Cognitive Variables*		AES-C	AES-B	AES-A
**Memory**	RAVLT immediate recall	−0.182	−0.025	−0.189
	RAVLT delayed recall	−0.157	−0.076	−0.179
	ROCF delayed recall	−0.228 *	−0.009	−0.229 *
**Praxis**	Apraxia constructional task	−0.215 *	−0.019	−0.181
	ROCF copy task	−0.311 **	−0.103	−0.248 *
**Executive Functions**	Phonological verbal fluency task	−0.114	−0.137	−0.191
	Semantic verbal fluency task	−0.209 *	−0.094	−0.240 *
	Stroop test-interference task	−0.529 **	−0.238 *	−0.330 **
	IML	−0.512 **	−0.332 **	−0.376 **
	CDT	−0.254 *	−0.152	−0.510 **
	TMT: B-A	0.237 *	0.194	0.299 *
**Reasoning**	RCMP	−0.269 *	−0.175	−0.304 *
***Behavioral Variable***				
**Depression**	HDRS	0.257 *	0.303 *	0.288 *

*, <0.05. **, 0.001 after Bonferroni correction are reported in bold. Note. MMSE: Mini-Mental State Examination; RAVLT: Rey Auditory Verbal Learning Test; ROCF: Rey–Osterrieth Complex Figure Test; IML: Inverse Motor Learning Test; CDT: Clock Drawing Test; TMT: Trail Making Test; RCPM: Raven’s Colored Progressive Matrices; AES-C: Apathy Evaluation Scale, a sub-scale score of cognitive apathy symptoms; AES-B: Apathy Evaluation Scale, a sub-scale score of behavioral apathy symptoms; AES-A: Apathy Evaluation Scale, a sub-scale score of affective apathy symptoms; HDRS: Hamilton Depressive Rating Scale.

## Data Availability

Data are available on request.
